# Global Transcriptomic Analysis of the Response of *Corynebacterium glutamicum* to Vanillin

**DOI:** 10.1371/journal.pone.0164955

**Published:** 2016-10-19

**Authors:** Can Chen, Junfeng Pan, Xiaobing Yang, Chenghao Guo, Wei Ding, Meiru Si, Yi Zhang, Xihui Shen, Yao Wang

**Affiliations:** 1 State Key Laboratory of Crop Stress Biology for Arid Areas and College of Life Sciences, Northwest A&F University, Yangling, Shaanxi 712100, China; 2 Department of Biochemistry and Molecular Biology, College of Life Sciences, Northwest A&F University, Yangling, Shaanxi 712100, China; 3 College of Life Science and Agronomy, Zhoukou Normal University, Zhoukou, Henan 466001, China; 4 College of Life Sciences, Qufu Normal University, Qufu, Shandong 273165, China; 5 College of Life Sciences and State Key Laboratory of Crop Stress Biology for Arid Areas, Bioinformatics Center, Northwest A&F University, Yangling, Shaanxi 712100, China; National Renewable Energy Laboratory, UNITED STATES

## Abstract

Lignocellulosic biomass is an abundant and renewable resource for biofuels and bio-based chemicals. Vanillin is one of the major phenolic inhibitors in biomass production using lignocellulose. To assess the response of *Corynebacterium glutamicum* to vanillin stress, we performed a global transcriptional response analysis. The transcriptional data showed that the vanillin stress not only affected the genes involved in degradation of vanillin, but also differentially regulated several genes related to the stress response, ribosome/translation, protein secretion, and the cell envelope. Moreover, deletion of the *sigH* or *msrA* gene in *C*. *glutamicum* resulted in a decrease in cell viability under vanillin stress. These insights will promote further engineering of model industrial strains, with enhanced tolerance or degradation ability to vanillin to enable suitable production of biofuels and bio-based chemicals from lignocellulosic biomass.

## Introduction

As a kind of new and renewable energy resources, biomass energy has rich reserves in the world [1]. The utilization of biomass energy provides a new option to cope with energy crisis that people faced to in the near future [2]. As important biomass energy resources, lignocellulose materials are potential sources for biofuels and other bio-based chemicals production [1–5]. At present, before these materials being applied into industrial production in a large scale, a series of problems need to be solved. One of those problems is that certain by-products (such as furan derivatives, weak acids, and phenolic compounds) showed up after the pretreatment of lignocellulose, which inhibit growth and fermentation of the industrial strains [6]. Vanillin is considered as one of the major inhibitors of phenolic compounds from pretreatment of lignocellulose, because it inhibits fermentation of microorganisms at very low concentrations [7]. So the studies on the tolerance and degradation to vanillin for robust strains become very important.

As a Gram-positive bacterium with high G+C content, *Corynebacterium glutamicum* is traditionally well known as a workhorse for the industrial production of various amino acids, and recent studies also explored it as production platforms for various chemicals, materials and fuels, such as the bio-based butanol and ethanol, the diamines cadaverine and putrescine, the sugar alcohol xylitol, gamma-amino butyric acid, polyhydroxybutyrate, pyruvate, lactate, 2-ketoisovalerate, 2-ketoglutarate and succinate [8, 9]. *C*. *glutamicum* is able to utilize a large number of lignocellulosic materials derived aromatic compounds (such as vanillin, ferulic acid, phenol, benzoate, 4-hydroxybenzoate, 4-cresol, resorcinol, benzyl alcohol, 2,4-dihydroxybenzoate, 3,5-dihydroxytoluene, etc.) for growth [10–17]. The extraordinary capability of *C*. *glutamicum* in assimilation of aromatic compounds as an alternative source to sugars makes it a unique advantage in utilizing lignocellulosic hydrolysates as sustainable resources in industrial fermentation [16].

Studies on the capability of microbe to detoxify and assimilate the vanillin as the carbon and energy resource had been taken in recent years [18, 19]. Genes involved in degradation of vanillin have been identified in *C*. *glutamicum*: *vdh* [16], *vanABK* [11] and *pcaHGBC* [13] gene clusters. However, although the inhibition of vanillin to several kinds of microorganisms (including yeast species, *Aspergillus* species, *Escherichia coli*, *Lactobacillus plantarum*, and *Listeria innocua*) [20, 21] has been evaluated, the adaption and tolerance to vanillin in *C*. *glutamicum* have not been investigated. Therefore, in this study, microarray analysis of the response of *C*. *glutamicum* to vanillin was conducted. Our work provides new insights into cellular response to vanillin stress that could be used to explore *C*. *glutamicum* as an efficient industry strain to convert sustainable lignocellulose to biofuels and bio-based chemicals in the future.

## Materials and Methods

### Bacterial strains and culture conditions

Bacterial strains and plasmids used in this study are listed in S1 Table. *E*. *coli* were grown aerobically on a rotary shaker (220 rpm) at 37°C in Luria-Bertani (LB) broth or on LB plates with 1.5% (wt/vol) agar. *C*. *glutamicum* strains were routinely grown in LB medium or in mineral salts medium supplemented with 0.05 g l^-1^ of yeast extract to meet the requirement of vitamins for the strains on a rotary shaker at 30°C [10]. Plasmid pXMJ19 was transformed into *C*. *glutamicum* RES167 wild type (WT), a restriction-deficient strain derived from *C*. *glutamicum* strain ATCC 13032, by electroporation for construction of WT(pXMJ19). For electroporation of *C*. *glutamicum*, brain heart broth with 0.5 M sorbitol (BHIS) medium was used. Cell growth was monitored by measuring absorbance at 600 nm (*A*_*600*_). Antibiotics were added at the following concentrations when needed: kanamycin, 50 μg ml^-1^ for *E*. *coli* and 25 μg ml^-1^ for *C*. *glutamicum*; nalidixic acid, 40 μg ml^-1^ for *C*. *glutamicum*; chloramphenicol, 20 μg ml^-1^ for *E*. *coli* and 10 μg ml^-1^ for *C*. *glutamicum* [22].

### Sensitivity Assays to vanillin

To test the susceptibility of *C*. *glutamicum* strains to vanillin, overnight cell cultures were diluted 100-fold with fresh LB medium and exposed to 90 mM vanillin for 40 min at 30°C with shaking. The cultures were serially diluted and plated onto LB agar plates and then the survival percentage was calculated as [(CFU ml^-1^ with stress)/(CFU ml^-1^ without stress)]×100 [23, 24]. All assays were performed in triplicate.

### Measurement of intracellular reactive oxygen species (ROS) levels

Intracellular ROS levels were measured using the fluorogenic probe 2’,7’-dichlorofluorescein diacetate (DCFH-DA) as described [25, 26], with the following modifications. Cells grown aerobically (OD_600_ = 1.6) were collected, washed and resuspended in PBS (pH 7.4) prior to preincubation with 2 μM DCFH-DA at 28°C for 20 min. Vanillin at indicated concentrations were added to these mixtures and incubated for another 40 min. After that, cells were washed two times with PBS, centrifuged, and resuspended in PBS. The fluorescence intensity was measured using a spectro-max spectrofluorimeter (excitation, 495 nm; emission, 521 nm).

### Validation of Microarray data by quantative real-time PCR (qRT-PCR)

The expression levels of 12 representative genes were examined by qRT-PCR to validate the microarry data. The primers for qRT-PCR were designed using Primer 5 (S2 Table). The cDNA synthesis was conducted using PrimeScript RT reagent Kit with gDNA Eraser (TaKaRa, Japan). qRT-PCR was conducted on BioRad CFX96 Real-Time System using SYBR Premix Ex Taq (TaKaRa). For each gene/sample combination, three replicate reactions were carried out. In addition, the 16 S rDNA gene was chosen as a reference gene. The qRT-PCR results were processed by “Bio-Rad CFX Manager 3.1” and gene expression ratios from the qRT-PCR were log_2_ transformed.

### Microarray experiments

The *C*. *glutamicum* DNA microarrays were custom-designed using the Agilent eArray 5.0 program according to the manufacturer’s recommendations (Agilent Technologies, Santa Clara, CA, US). The chip specification was 8×15K (design ID: 045822). Samples were collected during the mid-logarithmic growth phase in minimal medium with added glucose (control sample 100 mM) or vanillin as the sole carbon source (3 mM), respectively. Total RNA was extracted using TRIzol Reagent (Life Technologies, Carlsbad, CA, USA). Total RNA was amplified and labeled using the Low Input Quick Amp Labeling Kit, Two-Color (Agilent Technologies). Labeled cRNAs were purified using an RNeasy mini kit (Qiagen, GmBH, Germany). Each slide was hybridized with 300 ng Cy3/Cy5-labeled cRNA using the Gene Expression Hybridization Kit (Agilent Technologies) in a hybridization oven (Agilent Technologies). After 17 h of hybridization, slides were washed in staining dishes (Thermo Shandon, Waltham, MA, US) with a Gene Expression Wash Buffer Kit (Agilent Technologies). Slides were scanned using an Agilent Microarray Scanner (Agilent Technologies) with the default settings: dye channel, red & green; scan resolution, 5 μm; 16-bit. Data were extracted using the Feature Extraction software version 10.7 (Agilent Technologies). Raw data were normalized using Lowess (locally weighted scatter plot smoothing) algorithm in the Gene Spring Software version 11.0 (Agilent Technologies). Experiments were repeated four biological replicates in each condition. Differentially expressed genes were selected with p<0.01 by T-test methods. The fold changes of differentially expressed genes were log_2_ transformed. The microarray data has been deposited in NCBI Gene Expression Omnibus (GEO) database (accession number: GSE85949).

## Results and Discussions

### Overview of microarray analysis

**Fig 1:**
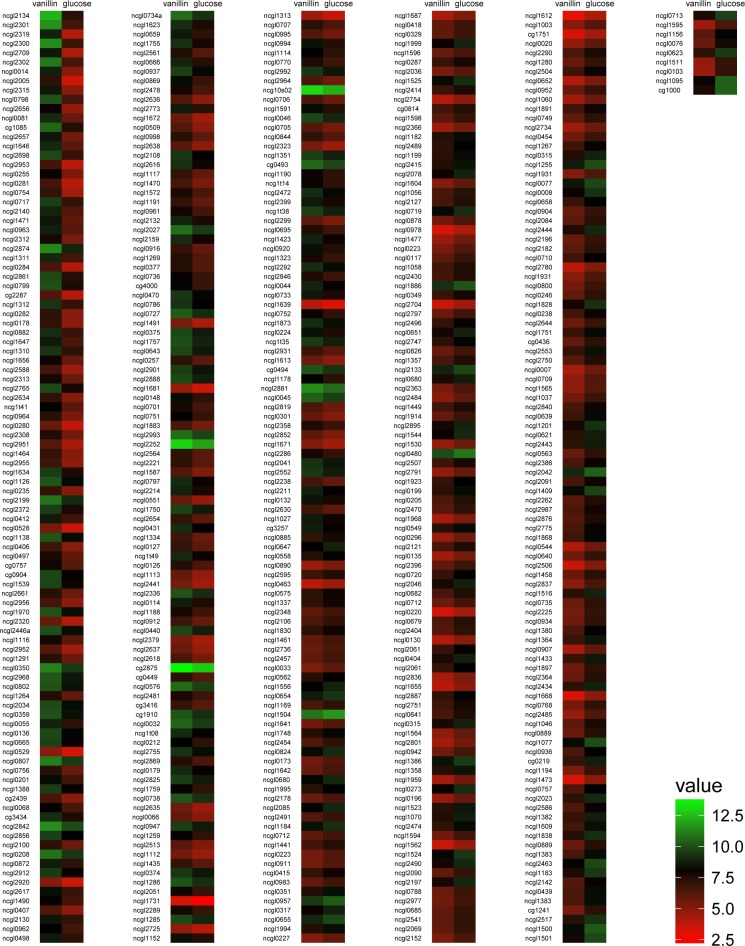
Microarray heat map of differential transcription of genes involved in response of *C*. *glutamicum* to vanillin. Heat map was generated using R program (version:i386 3.2.3). Red and green indicated lower or higher expression, respectively.

**Fig 2:**
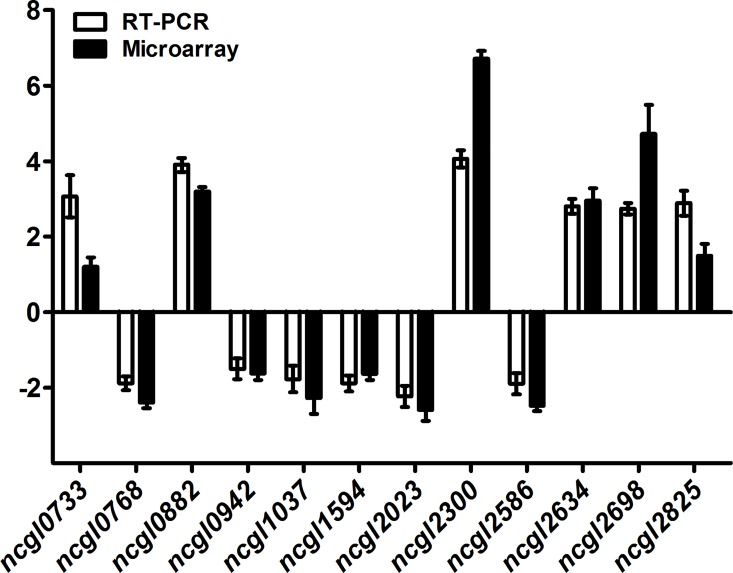
Validation of microarray results by qRT-PCR. Twelve representative genes were evaluated for validation of the microarray data using qRT-PCR. White bars show the mean log_2_-transformed fold changes of qRT-PCR from three biological replicates; Black bars represent the mean log_2_-transformed fold changes of microarray data from four biological replicates, and error bars indicate the standard deviations.

Gene expression patterns were assessed in the presence of vanillin and glucose as the sole carbon sources. To identify differentially expressed genes, bacteria in the mid-logarithmic growth phase were harvested for RNA extraction (S1 Fig) and further microarray experiment (hybridizations). The global analysis of differentially expressed genes was visualized by heat map (Fig 1). A total of 261 genes were up-regulated and 253 down-regulated. qRT-PCR of 12 representative genes was used to verify the microarray data. The log_2_-transformed mean values of qRT-PCR from three biological replicates for each gene were conformable to the log_2_-transformed fold changes of microarray data from four biological replicates in the microarray data (Fig 2).

### Further analysis of microarray data

**Fig 3:**
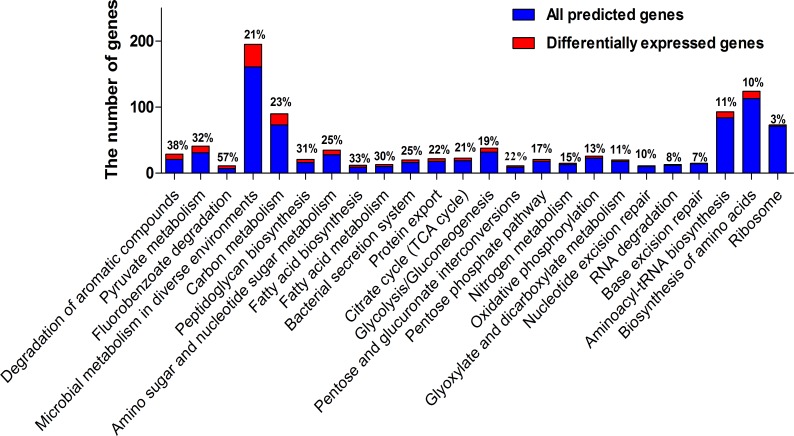
KEGG pathway analysis of differentially expressed genes. Summary of the number of differentially expressed genes in each KEGG pathway. The percentage of the differentially expressed genes account for the predicted genes are shown above the bars.

**Fig 4:**
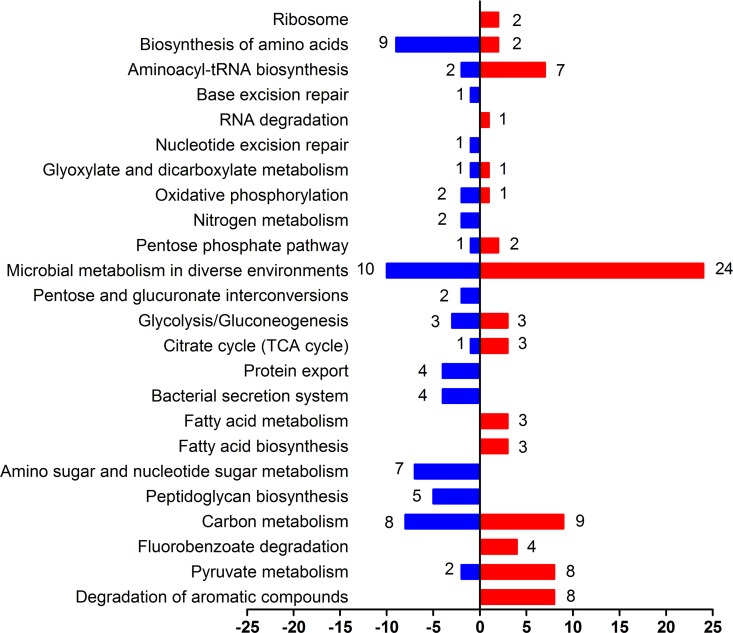
Differentially expressed genes (vanillin vs. glucose). The red and blue bars represent up- and down-regulated genes, respectively, and the numeric labels represent the number of genes with that function pathway.

We next identified the functions of the differentially expressed genes by Kyoto Encyclopedia of Genes and Genomes (KEGG) pathway analysis (Figs 3 and 4). Four kinds of pathways were interested to us: degradation of aromatic compounds, biosynthesis of amino acids, ribosome and bacterial secretion system.

#### Differentially expressed genes related to vanillin degradation

The vanillin generated by lignocellulose pretreatment can influence the growth and reduce production of microbial cells. However, bacteria such as *C*. *glutamicum* can adapt to the presence of this compound and utilize it as the sole carbon and energy source for growth [13]. *C*. *glutamicum* cells can survive by degrading vanillin, therefore, this degradation pathway was evaluated.

Our microarray data showed that *vanA* and *vanB* were up-regulated (Table 1). *vanK*, a major facilitator superfamily permease was up-regulated. Interestingly, *vanR*, a regulator to *vanABK* was up-regulated. But it was reported that VanR negatively regulates expression of the *vanABK* genes [27]. According to the previous study, *vanR* is transcribed leaderless [27], so generally the expression of *vanR* does not have great change on different situation. Nevertheless, the direct interaction of VanR with its effector vanillate, generated from degradation of vanillin, leaded to the deactivation of VanR [27], which may be the reason for the 1.2 fold change of *vanR* expression in our experiment. However, the *vanABK* genes were all up-regulated signally in the presence of 3 mM vanillin, which indicated the vanillin was catalyzed, and expression of *vanABK* genes was not fully inhabited by VanR.

**Table 1:** Differentially expressed genes related to vanillin degradation.

Locus tag	Gene name	Gene description	Fold change^a^	p-value^b^
*ncgl2299*	*vanR*	transcriptional regulator	1.20	0.004
*ncgl2300*	*vanA*	ferredoxin subunits of nitrite reductase and ring-hydroxylating dioxygenase	6.64	0.004
*ncgl2301*	*vanB*	flavodoxin reductase 1	6.94	0.004
*ncgl2302*	*vanK*	major facilitator superfamily permease	6.29	0.003
*ncgl2315*	*pcaH*	protocatechuate 3,4-dioxygenase subunit beta	5.24	0.007
*ncgl2313*	*pcaB*	3-carboxy-*cis*, *cis*-muconate cycloisomerase	3.00	0.009
*ncgl2312*	*pcaC*	4-carboxymuconolactone decarboxylase	3.58	0.002

^a^Log_2_-based expression ratio between glucose- and vanillin-grown cells.

^b^Microarray significance determined by p-value (p < 0.01).

*vanAB*, which encodes vanillate demethylase, catalyzes the conversion of vanillate to protocatechuate [11]. *pcaH*, which encodes one subunit of protocatechuate 3,4-dioxygenase was up-regulated (Table 1). This enzyme catalyzes conversion of protocatechuate to β-carboxy-*cis*, *cis*-muconate by a ring-cleavage reaction [13]. *pcaB*, which encodes β-carboxy-*cis*, *cis*-muconate cycloisomerase was up-regulated and *pcaC* which encodes γ-carboxymuconolactone decarboxylase was up-regulated. These two enzymes above catalyze conversion of β-carboxy-*cis*, *cis*-muconate to β-ketoadipate enol-lactone [13]. Therefore, according to transcriptome-level data, *C*. *glutamicum* was capable of degrading vanillin.

#### Differentially expressed genes related to the stress response

**Fig 5:**
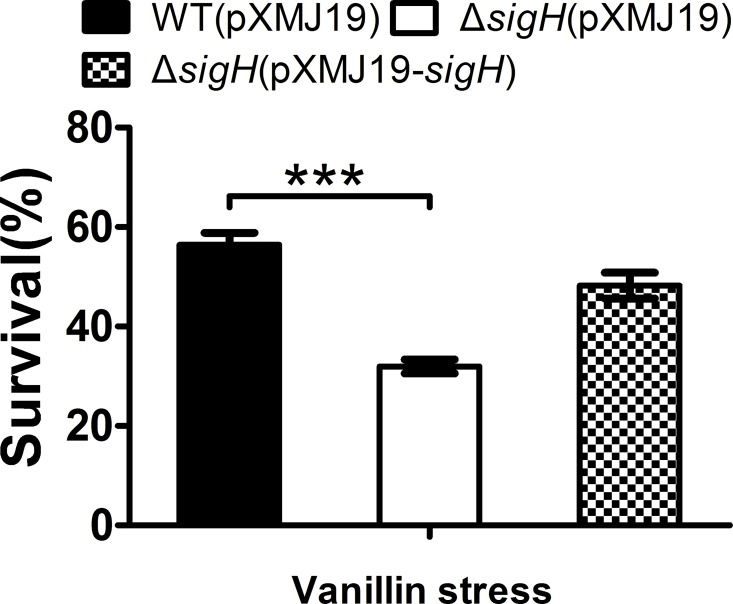
Δ*sigH* mutant was highly sensitive to vanillin stress compared to WT. Survival of the *C*. *glutamicum* WT(pXMJ19), Δ*sigH*(pXMJ19), and Δ*sigH*(pXMJ19-*sigH*) strains was assessed after exposure to vanillin (90 mM) for 40 min. Mean values with standard deviations (error bars) from at least three replicates are shown. ***: *P*≤0.001.

The extracytoplasmic function (ECF) σ factors have been identified in many species, and its regulation mechanisms had been studied in recent studies [28, 29]. *C*. *glutamicum* ATCC13032 has seven σ factor-encoding genes: *sigA*, *sigB*, *sigC*, *sigD*, *sigE*, *sigH*, and *sigM* [28, 30]. And *sigH* has been reported to take part in the heat stress or oxidative stress and regulate functional protein expressions to cope with stress conditions [28]. From our transcriptome data, *sigH* was up-regulated by vanillin stress (Table 2). Moreover, the *sigH* mutant was more sensitive to vanillin stress (90 mM) than was the WT strain, while the complemented strain had a survival rate similar to that of the WT (Fig 5).

**Table 2:** Differentially expressed genes related to the stress response.

Locus tag	Gene name	Gene description	Fold change^a^	p-value^b^
*ncgl0733*	*cgl0767*	RNA polymerase sigma factor RpoE/*sigH*	1.16	0.008
*ncgl2825*	*cgl2926*	methionine sulfoxide reductase A	1.36	0.005
*ncgl2842*	*cgl2943*	universal stress protein E	2.21	0.002
*ncgl2755*	*cgl2853*	universal stress protein	1.38	0.008
*ncgl2955*	*cgl3060*	oxidoreductase	2.76	0.003
*ncgl1886*	*cgl1961*	phage shock protein A	-1.34	3.50E-04
*ncgl0786*	*cgl0820*	cold shock protein	1.69	0.006
*ncgl2478*	*cgl2567*	dithiol-disulfide isomerase	1.96	0.009

^a^Log_2_-based expression ratio between glucose- and vanillin-grown cells.

^b^Microarray significance determined by p-value (p < 0.01).

**Fig 6:**
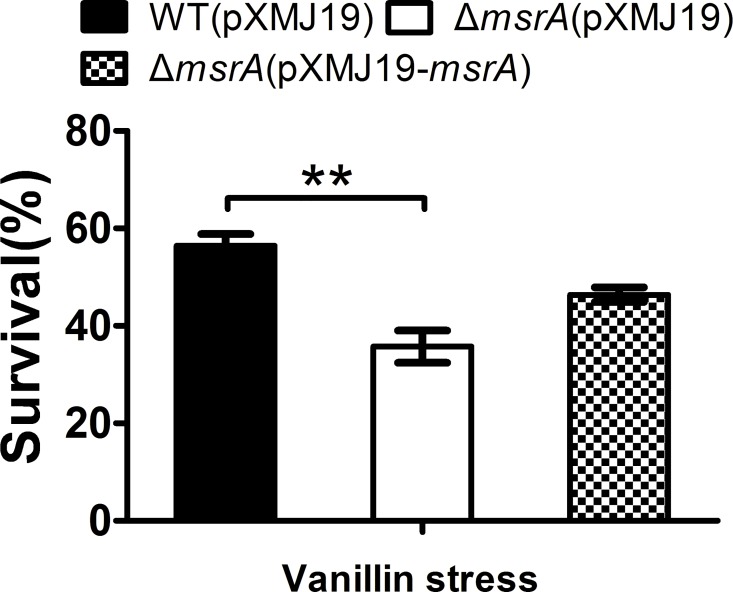
Δ*msrA* mutant was highly sensitive to vanillin stress compared to WT. Survival of the *C*. *glutamicum* WT(pXMJ19), Δ*msrA*(pXMJ19), and Δ*msrA*(pXMJ19-*msrA*) strains was assessed after challenge with vanillin (90 mM) for 40 min. Mean values with standard deviations (error bars) from at least three replicates are shown. **: *P*≤0.01.

**Fig 7:**
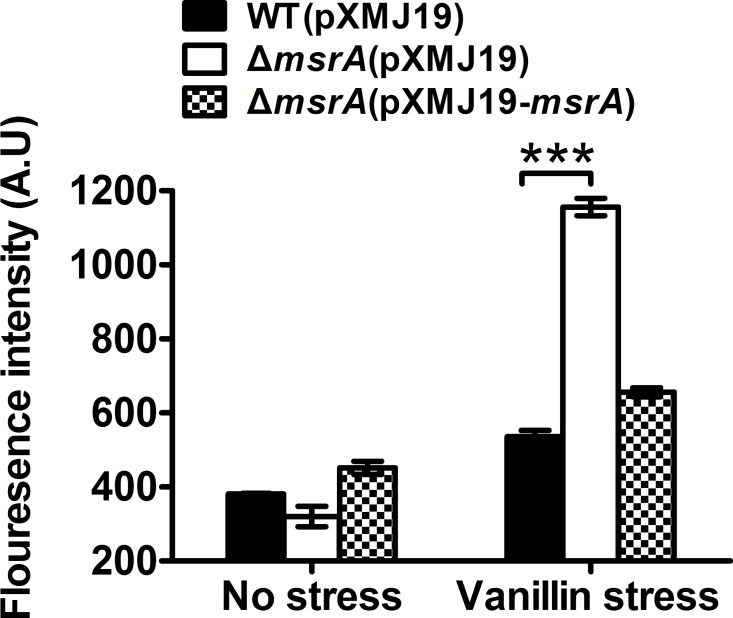
A mutant lacking MsrA exhibited increased ROS production under vanillin stress. A quantitative assay of intracellular ROS under vanillin stress was performed. Mean values with standard deviations (error bars) from three replicates are shown. ***: *P*≤0.001. The ROS levels in the indicated *C*. *glutamicum* strains were measured by a DCFH-DA fluorescence assay after exposure to vanillin.

Environmental factors such as UV radiation, ionization radiation, or many chemical compounds that produce intracellular reactive oxygen species (ROS) can arise the level of oxidative stress [31]. Bacteria have evolved complex systems to protect them against oxidative stress [31, 32]. These systems involve enzymes such as catalase, superoxide dismutase, methionine sulfoxide reductase (MsrA), etc. [31]. MsrA is one important kind of antioxidant repair proteins [33]. And the studies on the functions and mechanisms of MsrA to the oxidative stress by many agents had been taken in our previous studies [34]. MsrA coding gene (*msrA*) was up-regulated by vanillin stress (Table 2). The mutant was more sensitive to vanillin stress (90 mM) than that of WT, and the complemented strain had a survival rate similar to that of the WT (Fig 6). To evaluate the function of MsrA in ROS reduction in the presence of vanillin stress, ROS levels were examined using DCFH-DA. As shown in Fig 7, the *msrA* mutant had significantly higher ROS levels than those of the WT after vanillin stress treatment. Moreover, ROS levels in the *msrA* mutant could be restored by complementation to the similar levels in the WT (Fig 7).

The universal stress proteins in *E*. *coli* are induced by the stress of various environmental factors [35]. And in this study, *ncgl2842* and *ncgl2755*, encoding universal stress proteins, were up-regulated (Table 2), which may improve the ability to resistance to vanillin in *C*. *glutamicum*.

Bacteria have several proteins to degrade damaged DNA/proteins or protect functional proteins to maintain their metabolism [36]. Protein disulfide isomerase (PDI) interchanges thiol-disulfide that involve the reduction, rearrangement or formation of protein disulfide bonds [36]. In nascent proteins, PDI is important for disulfide bond formation and correct folding [36]. *ncgl2478*, which encodes a dithiol-disulfide isomerase in *C*. *glutamicum*, was up-regulated (Table 2) and this isomerase could protect proteins from further damage by vanillin stress.

There is a complicated stress response network in the cells [37]. More than one stress can be responded by the same system, and protect cells from a certain stress may bed several systems working together. [37]. Temperature induced stress is important for adaptation to environmental changes to living beings [37]. The cold shock protein A (CspA) is responded to many stress conditions: osmotic stress, inhibition of replication, starvation, UV sensitivity, freezing conditions, etc. [37]. From the microarray data, the cold shock protein A (*ncgl0786*) was up-regulated under the stress of vanillin in order to protect functional proteins under vanillin stress (Table 2).

The phage-shock-protein (Psp) system is induced by extracytoplasmic stress that can maintain the force of proton-motive, reduce cell energy status, maintain the integrity of cytoplasmic membrane and affect protein export [38]. However, in the Gram-negative Enterobacteriaceae: *Salmonella enterica* serovar Typhimurium, *E*. *coli*, and *Yersinia enterocolitica*, the Psp response has been most studied [39]. In this study, the phage shock protein A gene (*ncgl1886*) of *C*. *glutamicum* as one kinds of important Gram-positive model strain, was down-regulated under the vanillin stress (Table 2), which may relate to the stress response to vanillin.

#### Differentially expressed genes related to ribosome/translation

It has been reported that vanillin inhibits translation in *Saccharomyces cerevisiae* [7]. Vanillin can increase cytoplasmic messenger ribonucleoprotein (mRNP) granules and affect the large ribosomal subunit in *S*. *cerevisiae* [7].

In this study, we found several genes related to ribosome (such as *rpmH*, *tsnR*, *rpsF*) were up regulated by the affection of vanillin according to the transcriptome data (Table 3). And certain ribonuclease genes related to translation (such as *rnpA*, *rnc*, and *rph*) were differentially expressed (Table 3). Therefore, these results above indicated that vanillin did affect the ribosome or translation in *C*. *glutamicum*.

**Table 3:** Differentially expressed genes related to ribosome/translation.

Locus tag	Gene name	Gene description	Fold change^a^	p-value^b^
*ncgl2446a*	*-*	50S ribosomal protein L36	2.45	0.009
*ncgl2993*	*rpmH*	50S ribosomal protein L34	1.57	9.44–04
*ncgl1334*	*tsnR*	23S ribosomal RNA methyltransferase	1.51	4.53E-04
*ncgl2881*	*rpsF*	30S ribosomal protein S6	1.11	0.008
*ncgl2992*	*rnpA*	ribonuclease P	1.27	0.002
*ncgl1994*	*rnc*	ribonuclease III	-1.20	0.006
*ncgl2415*	*rph*	ribonuclease PH	-1.28	1.98E-06

^a^Log_2_-based expression ratio between glucose- and vanillin-grown cells.

^b^Microarray significance determined by p-value (p < 0.01).

#### Differentially expressed genes related to secretion protein

The Tat pathway and Sec pathway are two important kinds of protein secretion pathways in *C*. *glutamicum* [40]. Folded proteins are translocated by the Tat pathway, which is an alternative secretion pathway; unfolded proteins are translocated by the Sec pathway [40]. The *secD* gene which is one subunit of Sec pathway was down-regulated (Table 4). And two genes *tatB* and *tatC*, which belong to the subunits of Tat pathway, were down-regulated (Table 4). Many secretion proteins were differentially expressed from the analysis of microarray data (Table 4). The effects and mechanisms of vanillin stress to protein secretion in *C*. *glutamicum* need to be further studied.

**Table 4:** Differentially expressed genes related to secretion protein.

Locus tag	Gene name	Gene description	Fold change^a^	p-value^b^
*ncgl1594*	*secD*	preprotein translocase subunit SecD	-1.64	0.006
*ncgl1433*	*tatC*	Sec-independent protein secretion pathway component TatC	-2.34	0.002
*ncgl1077*	*tatB*	sec-independent protein translocase protein TatB	-2.41	0.002
*ncgl2661*	*cgl2757*	putative secreted protein	2.48	0.002
*ncgl0136*	*cgl0139*	putative secreted protein	2.36	0.003
*ncgl0872*	*cgl0909*	secreted protein	2.14	1.26E-05
*ncgl2912*	*cgl3015*	putative secreted protein	2.14	0.002
*ncgl0623*	*cgl0651*	putative secreted protein	-3.22	0.001
*ncgl0757*	*cgl0791*	putative secreted protein	-2.48	9.27E-04
*ncgl2225*	*cgl2307*	putative secreted protein	-2.27	0.002
*ncgl2775*	*cgl2883*	putative secreted protein	-2.11	0.006

^a^Log_2_-based expression ratio between glucose- and vanillin-grown cells.

^b^Microarray significance determined by p-value (p < 0.01).

#### Differentially expressed genes related to the cell envelope

Like *M*. *tuberculosis*, the cell envelope of *C*. *glutamicum* has several layers: plasma membrane, thick peptidoglycan-arabinogalactan layer, mycomembrane, and top layer [41]. This can enhance tolerance to various stress conditions including vanillin stress.

Genes related to cell wall (such as *ncgl0995*, *ncgl1156*, *ncgl2108*, *ncgl0126*, *ncgl0652*, and *ncgl2750*) were differentially expressed under the stress of vanillin according to the transcriptome data (Table 5).

**Table 5:** Differentially expressed genes related to the cell envelope.

Locus tag	Gene name	Gene description	Fold change^a^	p-value^b^
*ncgl0995*	*cgl1039*	glycosyltransferase, probably involved in cell wall biogenesis	1.27	0.004
*ncgl1156*	*cgl1203*	UDP-N-acetylmuramyl pentapeptide phosphotransferase	-2.98	0.002
*ncgl2108*	*cgl2188*	cell wall-associated hydrolase	1.85	0.005
*ncgl0126*	*cgl0127*	N-acetylglucosaminyltransferase	1.50	0.004
*ncgl0652*	*cgl0682*	cell wall-associated hydrolase	-1.76	0.009
*ncgl2750*	*cgl2847*	UDP-glucose 6-dehydrogenase	-1.97	3.25E-05
*ncgl0103*	*cgl0104*	membrane protein	-3.45	0.002
*ncgl0680*	*cgl0710*	membrane protein	-1.12	0.002
*ncgl0824*	*cgl0858*	metalloendopeptidase-like membrane protein	-1.10	9.85E-04
*ncgl0014*	*cgl0015*	membrane protein	5.56	0.002
*ncgl2372*	*cgl2458*	putative membrane protein	2.66	0.003

^a^Log_2_-based expression ratio between glucose- and vanillin-grown cells.

^b^Microarray significance determined by p-value (p < 0.01).

A previous study on the way of action of vanillin against *L*. *innocua*, *L*. *plantarum*, and *E*. *coli* suggested that it is an important membrane active compound [21]. From our microarray data, certain membrane protein genes were differentially expressed (Table 5).

#### Differentially expressed genes encoding master regulators

The transcription regulators play important roles in the metabolism and stress resistance processes for bacteria. The roles of various regulators in *C*. *glutamicum* were studied in recent years [42]. And we found two genes: *ramA* (*ncgl2472*) and *sigD* (*ncgl0575*) encoding master regulators were differentially expressed in our microarray data [42]. *ramA* was up-regulated 1.20-fold, which can activate the genes related to acetate metabolism, aconitase gene *acn*, glyceraldehyde-3-phosphate dehydrogenase gene *gapA*, et al [43]. This may show the different mechanisms of carbon metabolism between glucose and vanillin in *C*. *glutamicum*. Except the *sigH*, another gene encoding ECF σ factor SigD was down-regulated 1.03-fold in our microarray data. It was reported that SigD is induced by cold shock and is necessary to full virulence in *Mycobacterium tuberculosis* [44, 45]. In *Clostridium difficile*, SigD as a regulator can positively regulate the expression of toxin [46]. However, the function of SigD in *C*. *glutamicum* is not clear by now. It may play a role in the response to vanillin in *C*. *glutamicum*.

## Conclusions

**Fig 8:**
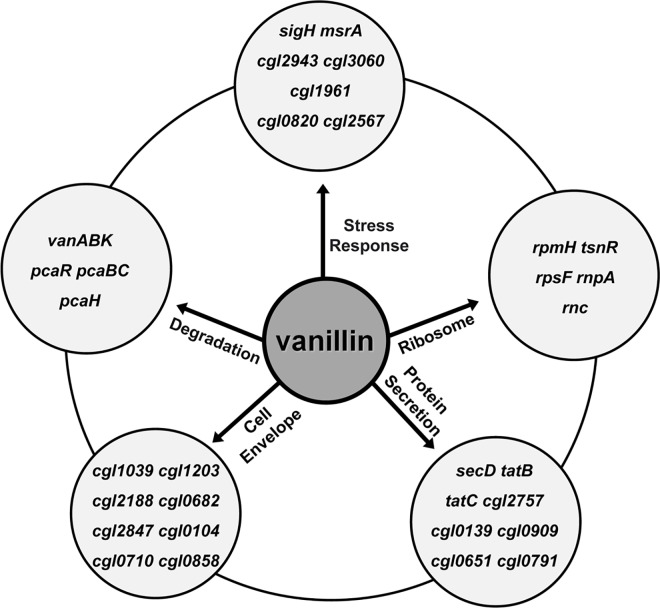
Response of *C*. *glutamicum* to vanillin. Schematic diagram of the genes involved in the response of *C*. *glutamicum* to vanillin stress.

The mechanisms of tolerance to vanillin inhibitor generated by lignocellulose pretreatment of *C*. *glutamicum* were as follows. First, *C*. *glutamicum* was able to degrade vanillin. Second, the *C*. *glutamicum* cell envelope, which has complex structures, has a greater protective effect than other microbes. Third, genes related to stress response were differentially expressed under vanillin stress conditions, which could reduce the damage to *C*. *glutamicum* cells. Fourth, ribosome/translation and protein secretion genes were differentially expressed to cope with the vanillin stress (Fig 8). The *sigH* and *msrA* mutants were more sensitive to vanillin stress. Therefore, *C*. *glutamicum* can degrade vanillin to reduce the damage caused. And moreover, this microorganism possesses defense and damage repair mechanisms.

To date, this is the first report of a transcriptomic analysis of the response to vanillin by *C*. *glutamicum*. The results provide insights into the mechanisms of *C*. *glutamicum* adaption and tolerance to vanillin, an important lignocellulose-derived inhibitor. This provides a theoretical basis for the engineering of industrial microorganisms tolerant to vanillin and makes facile production of biofuels and bio-based chemicals from lignocellulosic biomass in the future.

## Supporting Information

S1 Fig Growth curves of *C*. *glutamicum* using glucose or vanillin, respectively. Growth of *C*. *glutamicum* on mineral salts medium containing 100 mM glucose (A) and 3 mM vanillin (B). Arrows indicate the sampling points for microarray analysis.
(TIF)

S1 Table Bacterial strains and plasmids used in this study. (DOC)

S2 Table Primers used in this study. (DOC)
