# Supplementary figures and images for: Global Transcriptomic Analysis of the Response of Corynebacterium glutamicum to Vanillin
Source: PLoS One. 2016 Oct 19;11(10):e0164955. doi: 10.1371/journal.pone.0164955 (PMC5070772; doi:10.1371/journal.pone.0164955)

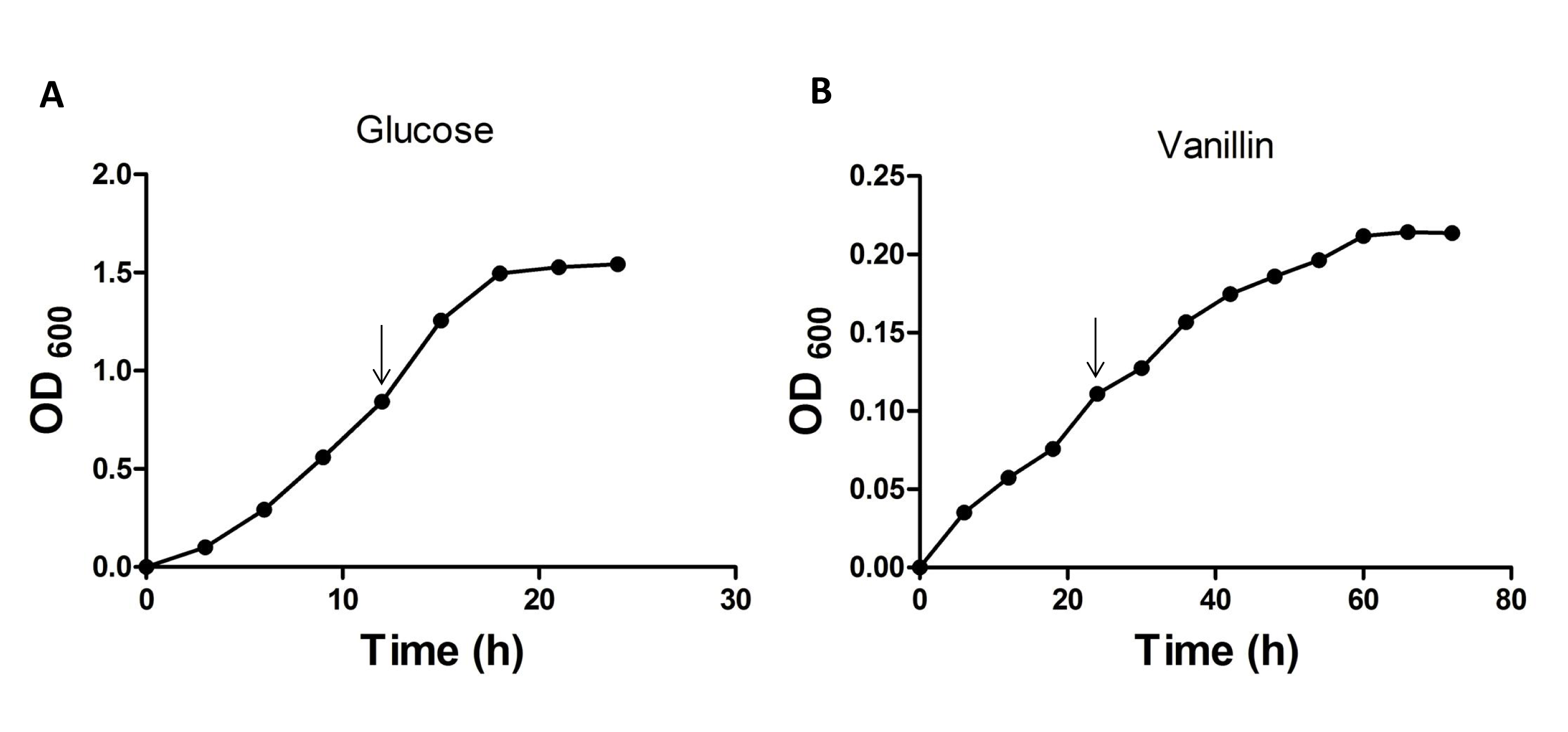

Supplement: S1 Fig — Growth of C. glutamicum on mineral salts medium containing 100 mM glucose (A) and 3 mM vanillin (B). Arrows indicate the sampling points for microarray analysis. (TIF) [file pone.0164955.s001.tif]
