# Supplementary material for: Global Transcriptomic Analysis of the Response of Corynebacterium glutamicum to Vanillin
Source: PLoS One. 2016 Oct 19;11(10):e0164955. doi: 10.1371/journal.pone.0164955 (PMC5070772; doi:10.1371/journal.pone.0164955)
Supplement: S1 Table — (DOC) [file pone.0164955.s002.doc]

**S1Table.** Bacterial strains and plasmids used in this study

| **Strains or plasmids** | **Relevant genotype description** | **References** |
| --- | --- | --- |
| **Strains** | | |
| ***C. glutamicum*** | | |
| RES167（WT） | Restriction-deficient mutant of ATCC13032, Δ(*cglIM-cglIR-cglIIR*) | Tauch et al. 2002 |
| WT(pXMJ19) | RES167 containing pXMJ19 vector | This study |
| Δ*sigH* | *sigH* deleted in RES167 | Si et al. 2014 |
| Δ*msrA* | *msrA* deleted in RES167 | Si et al. 2014 |
| Δ*sigH*(pXMJ19-*sigH*) | Complement of *sigH* in Δ*sigH* mutant | Si et al. 2014 |
| Δ*msrA*(pXMJ19-*msrA*) | Complement of *msrA* in Δ*msrA* mutant | Si et al. 2014 |
| ***E. coli*** | | |
| JM109 | recA1 supE44 endA1 hsdR17 gyrA96 relA1 thi Δ(lac-proAB)F′(traD36 proABlacIq lacΔZM15) | Stratagene |
| **Plasmids** | | |
| pXMJ19 | Shuttle vector (*P_tac_ lacI^q^ pBL1 oriV_C. glutamicum_* pK18 *oriV_E. coli_*) | Jakoby et al. 1999 |

**References**

1. Tauch A, Kirchner O, Loffler B, Gotker S, Pühler A, Kalinowski J. Efficient electrotransformation of *Corynebacterium diphtheriae* with a mini-replicon derived from the plasmid pGA1. Curr. Microbiol. 2002;45:362–367.

2. Si MR, Long MX, Chaudhry MT, Xu Y, Zhang P, Zhang L, et al. Functional characterization of *Corynebacterium glutamicum* mycothiol S-conjugate amidase. PLoS One. 2014;9: e115075.

3. [Jakoby](http://link.springer.com/article/10.1023/A:1008968419217#author-details-1) M, [Ngouoto-Nkili](http://link.springer.com/article/10.1023/A:1008968419217#author-details-2) CE, [Burkovski](http://link.springer.com/article/10.1023/A:1008968419217#author-details-3) A. Construction and application of new *Corynebacterium glutamicum* vectors. [Biotechnol Te](http://www.medsci.cn/sci/submit.do?id=235e1147)ch. 1999; 13(6): 437–441.
