# Supplementary material for: Global Transcriptomic Analysis of the Response of Corynebacterium glutamicum to Vanillin
Source: PLoS One. 2016 Oct 19;11(10):e0164955. doi: 10.1371/journal.pone.0164955 (PMC5070772; doi:10.1371/journal.pone.0164955)
Supplement: S2 Table — (DOC) [file pone.0164955.s003.doc]

**S2 Table. Primers used in this study**

| **Primiers** | **5’-3’ sequence** |  |
| --- | --- | --- |
| 16s-RT-F | AGAACCTTACCTGGGCTTGA | For qRT-PCR |
| 16s-RT-R | CGCTCGTTGCGGGACTTA |  |
| *ncgl0733*-RT-F | CGGATGGTGGTTTATTATGC |  |
| *ncgl0733*-RT-R | TGTTCCTTCGCTACTTCCTTT |  |
| *ncgl0942*-RT-F | GATACAGTGCGCTACTGGTAC |  |
| *ncgl0942*-RT-R | CTCCTCCTGGGGATAAATT |  |
| *ncgl1594*-RT-F | CGTTGGTCAGGCAGGAATG |  |
| *ncgl1594*-RT-R | GCAACAGCGGTGTTCATTTC |  |
| *ncgl0882*-RT-F | TGGGAACCTATCCGAAGCC |  |
| *ncgl0882*-RT-R | CGCTGACCGAAATAAGTAAAGC |  |
| *ncgl0768*-RT-F | CATCGGGAAGAAGAAGGACA |  |
| *ncgl0768*-RT-R | TCAGCAAGCAAGACGAGTGG |  |
| *ncgl1037*-RT-F | TGCCCGCATCTTTG |  |
| *ncgl1037*-RT-R | GCTCCGCCATCTTCA |  |
| *ncgl2023*-RT-F | GGCATCACCTCTGACCACA |  |
| *ncgl2023*-RT-R | ACCAGCGTAAACTCCCACC |  |
| *ncgl2586*-RT-F | CGTAAAACCGCACCACACC |  |
| *ncgl2586*-RT-R | ATCGCCCTCCAAAACAATG |  |
| *ncgl2634*-RT-F | GCGTGAAAGCGAAATCT |  |
| *ncgl2634*-RT-R | GTAGGCAAGCGACAGCA |  |
| *ncgl2698*-RT-F | GGCTGGTCGAGTTTGGGTC |  |
| *ncgl2698*-RT-R | CAGGTTCTTGTTCTGCTGGTAG |  |
| *ncgl2300*-RT-F | ATAAGTTCCCAGGATTTGA |  |
| *ncgl2300*-RT-R | TCCAGCCTTTGCTACAC |  |
| *ncgl2825*-RT-F | CCGCCAGGGTAACGATGT |  |
| *ncgl2825*-RT-R | CCGATTTCCGTGGTGATTT |  |
